# Epidermal growth factor receptor (EGFR) mutations are exceptionally rare in thyroid transcription factor (TTF-1)-negative adenocarcinomas of the lung

**DOI:** 10.18632/oncoscience.72

**Published:** 2014-08-07

**Authors:** Neeta Somaiah, Mary Jo Fidler, Elizabeth Garrett-Mayer, Amy Wahlquist, Keisuke Shirai, Lela Buckingham, Thomas Hensing, Philip Bonomi, George R. Simon

**Affiliations:** ^1^ MD Anderson Cancer Center, Houston TX 77004; ^2^ Rush University Medical Center, Chicago, IL 60612; ^3^ Hollings Cancer Center, Medical University of South Carolina, Charleston, SC 29425; ^4^ North Shore University Health Systems/University of Chicago, Evanston, IL 60201.

**Keywords:** TTF-1, EGFR, NSCLC, adenocarcinoma

## Abstract

**Introduction:**

Approximately 70% of lung adenocarcinomas express TTF-1. EGFR mutations are present in 13-15% of Western adenocarcinoma patients. This paper investigates TTF1 as a negative predictor of mutant EGFR in lung adenocarcinomas.

**Results:**

In the pilot cohort (N = 301) two of 224 specimens positive for EGFR mutations had negative TTF-1 expression (sensitivity 99.1%, 95% confidence interval (CI) 96.8-99.9%). Estimated negative predictive values (NPV) for EGFR mutation prevalence rates of 13% and 15% are 99.5% (95% credible interval (CRI) 98.6%-99.9%) and 99.4% (CRI – 98.4%-99.9%). For EGFR mutation rates of 13% and 15%, using validation cohort data (211 patients), the estimated NPVs were 97% (95% CRI 92%-99%) and 96% (95% CRI 91%-99%).

**Methods:**

Formalin-fixed paraffin-embedded tumors from lung adenocarcinoma patients were analyzed for EGFR mutations by allele-specific PCR in the ‘pilot cohort’. TTF-1 status was documented as positive or negative. Negative predictive value (NPV) for a range of true prevalence of EGFR mutation (1%-50%) was estimated using Bayesian modeling. The hypothesis was validated in a separate ‘validation’ cohort using the same modeling.

**Conclusion:**

An overwhelming majority of TTF-1 negative adenocarcinomas will be negative for EGFR mutations. This finding allows for earlier initiation of chemotherapy in newly diagnosed TTF-1 negative adenocarcinomas of the lung with stage IV disease.

## INTRODUCTION

Distinctive genetic aberrations can induce the proliferation of cancer cells and render them vulnerable to therapeutic targeting by specific inhibitors. Inhibitors of the epidermal growth factor receptor (EGFR) are a prototypic example and have produced consistently high response rates in a subgroup of patients with non–small-cell lung cancer (NSCLC) with activating *EGFR* mutations [1, 2]. Certain patient and tumor characteristics such as adenocarcinoma histology, never-smoking status, female sex and East Asian origin, increase the probability of having EGFR mutations [3, 4]. The frequency of these mutations in lung adenocarcinomas for patients in the western hemisphere range between 10-18% compared to approximately 2% in other NSCLC histological subtypes [5-7].

TTF-1, encoded by the *NKx2* gene, is a homeobox transcription factor that is linked to normal lung development and function. Genomic profiling studies in lung cancer cell lines and tumors reveal frequent focal DNA amplification at 14q13.3 [8-11]. *TTF-1* amplified lung cancers, exhibit increased RNA and protein expression. However, increased TTF-1 protein expression is not necessarily associated with *TTF-1* amplification at the DNA level and occurs in different subsets of patients with distinct clinico-pathological and molecular features [12]. TTF-1 protein expression is prevalent in lung adenocarcinomas and is significantly associated with female gender, never-smoking status, presence of *EGFR* mutations and better overall survival [13-16]. *TTF-1* amplification, on the other hand, has been detected in both lung adenocarcinomas (18.9%) and squamous cell carcinomas (20.1%) and correlated significantly with the presence of *KRAS* mutations in the adenocarcinoma subset [8-10, 17]. TTF-1 protein expression (standard expression defined as greater than 5% of tumor cells with moderate (2+) or strong (3+) nuclear staining considered positive) is selectively found in adenocarcinomas of the lung and thyroid cancers and its practical diagnostic utility lies in differentiating adenocarcinomas from pulmonary or non-pulmonary origins. Studies have previously reported that tumors positive for *EGFR* mutations have a high frequency of TTF-1 positivity though the negative predictive value (NPV) for varying *EGFR* mutation prevalences have not been previously reported [18, 19]. We thus proceeded to prove the hypothesis that TTF-1 negative tumors will be wild type for EGFR mutations. The clinical utility of this finding is evident and would justify the earlier initiation of front line cytotoxic chemotherapy in TTF-1 negative advanced stage patients with adenocarcinomas of the lung.

## RESULTS

### The pilot cohort

Of the 301 lung adenocarcinomas samples 224 were positive for *EGFR* mutations (74%). Of the 224 mutant EGFR tumors, 119 had an exon 19 deletion, 84 had an exon 21 mutations (79 patients had L858R and 5 patients had L861Q), 14 had an exon 18 mutation (G719X) and 3 had a resistance mutation S768I in addition to a sensitizing mutation. Four were labeled as ‘deletions’ without further classification.

Only 2 (one exon 19 deletion and one L858R mutation) of the 224 specimens positive for EGFR mutations and 28 of the 77 EGFR wild-type patients were TTF-1 negative (Table 1). Thus the sensitivity is 99.1% (95% CI: 96.8-99.9%) and specificity is 36.4% (95% CI: 25.7-48.1%). For assumed EGFR mutation prevalence rates of 13% and 15%, the estimated NPVs are 99.5% (95% CRI: 98.6%-99.9%) and 99.4% (95% CRI: 98.4%-99.9%), respectively. Even for a prevalence of EGFR mutations as high as 30% the NPV is 98.5.

The estimated NPV with the 95% CRI for a range of EGFR mutation prevalence is shown in Figure 1A. From this model, the estimates of sensitivity and specificity were 98.8% (95% CRI 96.8% – 99.7%) and 36.4% (95% CRI: 26.3% – 47.4%), which are almost identical to the actual observed values noted above. Our analyses using different Beta priors provided very similar inference for true EGFR mutation rates of 13% and 15%: 99.7% and 99.6% for a Beta (0.005, 0.005) prior and 99.4% and 99.2% for a Beta (2, 2) prior.

**Table 1:** Distribution of samples by EGFR mutation and TTF-1 status in the pilot cohort

		EGFR	Status	
		Mutant	Wild Type	Total
**TTF-1**	Positive	222	49	271
	Negative	2	28	30
	Total	224	77	301

Sensitivity = 99.1% (95% CI: 96.8-99.9%)

NPV for EGFR mutation rates of 13% = 99.5% (95% CRI: 98.6%-99.9%)

NPV for EGFR mutation rates of 15% = 99.4% (95% CRI: 98.4%-99.9%).

(NPV = Negative Predictive Value)

### The validation cohort

In the validation cohort, 131 advanced lung adenocarcinoma patients with known EGFR mutation status and TTF-1 status were analyzed. Twenty-one patients with EGFR mutant tumors were identified, with 12 harboring an exon 19 deletion and 9 harboring a mutation in exon 21.

Only 1 of the 21 EGFR mutation positive specimens and 35 of the 110 EGFR wild-type specimens were TTF-1 negative (Table 2), yielding a sensitivity of 95% (95% CI: 76%-99.9%) and specificity of 32% (95% CI: 23%-41%). For true *EGFR* mutation rates of 13% and 15% the estimated NPVs are 97% (95% CRI: 90%-99%) and 96% (95% CRI: 88%-99%), respectively (Figure 1B), thus validating our findings in the pilot cohort.

**Table 2:** Distribution of samples by EGFR mutation and TTF-1 status in the validation cohort

		EGFR	Status	
		Mutant	Wild Type	Total
**TTF-1**	Positive	20	75	95
	Negative	1	35	36
	Total	21	110	131

Sensitivity = 95% (95% CI: 76%-99.9%)

NPV for EGFR mutation rates of 13% = 97% (95% CRI: 90%-99%)

NPV for EGFR mutation rates of 15% = 96% (95% CRI: 88%-99%).

(NPV = Negative Predictive Value)

## DISCUSSION

A favorable prognosis for TTF-1 (encoded by NKX2-1) in NSCLC has been documented in many retrospective studies [12-15]. Evaluation of metastatic lesions in mouse models show that TTF-1 is consistently depressed in metastatic versus non-metastatic lung adenocarcinoma [11]. This is potentially mediated by preservation of the epithelial phenotype as TTF1 transactivates e-cadherin and other tight junction molecules via promoter interaction [20-22]. In NKX2-1 negative mice models with *KRAS* mutations, reduced tumor volume was seen when TTF-1 expression was induced [11, 23]. The ability of the TTF-1 protein to confer a favorable prognosis, observed in cell line and xenograft studies, have been corroborated in clinical trials testing front line cytotoxic chemotherapy in patients with stage IV NSCLC [24].

The high negative predictive value exceeding 96% of TTF-1 for the presence of the activating *EGFR* gene mutation is intriguing. Cell line data from Yamaguchi et al show receptor tyrosine kinase-like orphan receptor 1 (ROR1) is highly upregulated after introduction of NKX2-1. ROR1 negatively regulates phosphoinositide 3-kinase (PI3K) and conversely ROR1 knockdowns enhance PI3K activity. ROR1 kinases activity was thought to be required to fully sustain downstream signaling of EGF stimulated EGFR. ROR1 signaling also decreased phosphorylation of phosphatase and tensin homolog (PTEN), and encouraged EGFR-ERBB3 interaction [25]. Though mutations in *EGFR* are thought to be a driver in adenocarcinomas, it is possible that *TTF-1* (being a transcription factor) facilitates the transcription of necessary genes to maintain survival signaling and evade apoptosis.

Interestingly, preliminary evidence suggests that there exists an association between the echinoderm microtubule-associated protein-like 4-anaplastic lymphoma kinase (EML4-ALK) translocations, another oncogenic driver, and TTF-1 status. TTF-1 positivity in ALK translocated tumors seems to be present approximately 70% of the time. The co-expression of p63 is also often seen in these tumors [26-28]. Currently, approximately 60% of lung adenocarcinomas have now been shown to have mutations (KIF5B-Ret, ROS1, NTRK1, HER2-Neu, to name a few) that may induce and drive these malignancies and the association of TTF-1 status and presence of these driver mutations remains to be elucidated [29-37].

Whether TTF-1 itself has a tumor suppressor or tumor-promoting function awaits further clarification. Multivariate analyses reveal that *TTF-1* amplification and copy number gain are independent predictors of poor survival in patients with NSCLC [12-14]. Indeed small interfering RNA (siRNA)-mediated knockdown of *TTF-1* in lung cancer cell lines with amplification led to reduced cell proliferation, manifested by both decreased cell-cycle progression and increased apoptosis indicating that *TTF-1* amplification is a lineage-specific oncogene in lung cancer [8-11]. Thus protein expression and DNA amplification of *TTF-1* have divergent implications in patients with lung cancer and a clearer understanding of this phenomenon would further clarify the significance of the association between certain driver mutations and the expression of the TTF-1 protein [32].

In this study the sensitivity and specificity for TTF1 and the presence of *EGFR* gene mutation was lower in the validation cohort than the pilot cohort. These differences may be accounted for by differences in *EGFR* gene mutation testing methods. The most common mutations associated with sensitivity to EGFR tyrosine kinase inhibitors are exon 19 deletions and by point mutations in exon 21 (L858R). These two mutations account for approximately 90% of all EGFR mutations [38]. In our pilot cohort, specimens were analyzed for all known EGFR mutations, and these two common account for 90% of the mutation. In our validation cohort, specimens were analyzed only for two common mutations, and conceivably, if the rare mutations were also included, the sensitivity and the NPV might have been higher.

Current guidelines recommend testing all non-squamous NSCLCs for *EGFR* mutations, and treating mutation positive patients with EGFR tyrosine kinase inhibitors (TKIs) first-line. Several randomized trials have validated this approach [39-43]. However, most institutions and community cancer centers depend on centralized CLIA-certified laboratories for testing. This entails sending tissue for analysis and waiting several weeks before EGFR mutation results become available, whereas TTF-1 status is conveniently available with the institutional pathology report. Our data demonstrates that patients with adenocarcinomas of the lung who are TTF-1 negative have at least a 96% chance of being *EGFR* wild type when the prevalence of EGFR mutations is approximately 15%. This allows for early initiation of front-line chemotherapy in patients with bronchogenic adenocarcinoma which is TTF-1 negative.

## METHODS

To test the above outlined hypothesis, data were acquired from tissue samples sent by treating medical oncologists to a central reference Clinical Laboratory Improvement Amendments (CLIA) certified laboratory (Response Genetics Inc., Los Angeles, CA) for *EGFR* mutation testing (Pilot cohort). Samples were reviewed for EGFR mutation status and TTF-1 status. Microdissected formalin-fixed paraffin-embedded tumors were used for *EGFR* mutation analysis by allele-specific polymerase chain reaction (PCR) to identify all known EGFR mutations (exon 18-21). TTF-1 status was based on IHC staining of the tumor specimen as recorded on the accompanying pathology report (required to be done in a CLIA certified clinical pathology laboratory). As the pilot data set is composed of specimens sent by physician referral, the pilot set was highly enriched for the presence of EGFR mutations. Adenocarcinoma specimens with known TTF-1 status were analyzed for any of the above identified *EGFR* mutations and coded as mutant vs. wild-type.

For the Validation cohort, stage IV lung adenocarcinoma patients treated with erlotinib at Rush University Medical Center, Chicago, IL and Evanston Hospital, Evanston, IL were included if there was sufficient tissue available for *EGFR* mutation testing and TTF-1 status was known. Patients' tumors were analyzed for only the two most common mutations that are associated with sensitivity to EGFR tyrosine kinase inhibitors (exon 19 deletion or exon 21 L858R point mutation) using single-strand conformation polymorphism (SSCP) and sequence-specific PCR. Tumors were labeled wild-type if these two common mutations were absent.

### Statistical considerations

Descriptive statistics were used to summarize characteristics of patients whose samples were included in the analysis. Sensitivity and specificity were calculated, representing the probability of a TTF-1 positive result given that an EGFR mutation is present, and the probability of a TTF-1 negative result given no evidence of the presence of an EGFR mutation, respectively. Exact 95% confidence intervals were calculated. Because the prevalence of the EGFR mutations in the pilot data group of lung adenocarcinomas was not reflective of the true prevalence in most clinical settings, the negative predictive value (NPV) generated from our pilot data would not provide an accurate estimate of the NPV of TTF-1 in a clinical setting in the US, where it has been shown that approximately 10-18% of patients with advanced stage lung adenocarcinomas have an EGFR mutation [5, 7]. However, NPV can be calculated as function of sensitivity (*Sens*) and specificity (*Spec*) for another assumed EGFR mutation prevalence (π) using Bayes rule:

$$\text{NPV}=\frac{\text{Spec}(1-\pi )}{\text{Spec}(1-\pi )+(1-\text{Sens})\pi }$$

We considered values of prevalence of EGFR from p = 0.01 to 0.50. Although estimation of NPV using the above relationship between NPV, sensitivity, specificity and prevalence is simple, the estimation of the precision of NPV requires statistical modeling. A Bayesian estimation approach was used where TTF-1 results were assumed to follow binomial distributions, based on EGFR status and *Beta(1,1)* priors were used for modeling sensitivity and specificity. *Beta(1,1)* is considered a “weak” or ‘flat” prior. WinBugs14, a Gibbs sampler software program, was used for model fitting. Separate models were fit to estimate NPV over a range of assumed true EGFR mutation prevalences (1% to 50% prevalence). From these posterior distributions, for each of the assumed prevalences, the median, 2.5**^th^** and 97.5**^th^** quintiles provide point estimates and 95% credible intervals for the NPV. CI refers to confidence intervals and CRI refers to credible intervals.

The Gibbs sampler was run multiple times with different starting values to ensure convergence of results. Trace plots were also examined. A burn-in of 50,000 iterations was used. Fifty thousand iterations were rerun where every 10**^th^** iteration was saved for generating posterior distributions. The Gibbs sampler was also rerun using a *Beta(0.005, 0.005)* prior (which favors very high and low probability values but is relatively flat for most values between 0.02 and 0.98) and a *Beta(2,2)* prior (which favors probability values near 0.50 relative to high and low values) to ensure the results were not sensitive to the choice of prior.

## CONCLUSION

Our findings that EGFR mutations are associated with TTF-1 expression have significant clinical and biological implications. Chemotherapy can be initiated in stage IV patients with TTF-1 negative adenocarcinoma of the lung, while their mutation testing is awaited. In capital constrained clinical practice settings, our findings will enable the more optimal utilization of both financial and personnel resources. Additionally study of TTF-1 related molecular pathways might provide new insights regarding cancer initiation, progression, and treatment for this relatively large group of non-small cell lung cancer patients.
